# Developing methylotrophic microbial platforms for a methanol-based bioindustry

**DOI:** 10.3389/fbioe.2022.1050740

**Published:** 2022-11-23

**Authors:** Hawaibam Birla Singh, Min-Kyoung Kang, Moonhyuk Kwon, Seon-Won Kim

**Affiliations:** ^1^ Division of Applied Life Science (BK21 Four), ABC-RLRC, PMBBRC, Gyeongsang National University, Jinju, South Korea; ^2^ Division of Life Science, ABC-RLRC, PMBBRC, Gyeongsang National University, Jinju, South Korea

**Keywords:** methylotrophs, methanol, microbial cell factories, bio-based chemical production, metabolic engineering, synthetic biology

## Abstract

Methanol, a relatively cheap and renewable single-carbon feedstock, has gained considerable attention as a substrate for the bio-production of commodity chemicals. Conventionally produced from syngas, along with emerging possibilities of generation from methane and CO2, this C1 substrate can serve as a pool for sequestering greenhouse gases while supporting a sustainable bio-economy. Methylotrophic organisms, with the inherent ability to use methanol as the sole carbon and energy source, are competent candidates as platform organisms. Accordingly, methanol bioconversion pathways have been an attractive target for biotechnological and bioengineering interventions in developing microbial cell factories. This review summarizes the recent advances in methanol-based production of various bulk and value-added chemicals exploiting the native and synthetic methylotrophic organisms. Finally, the current challenges and prospects of streamlining these methylotrophic platforms are discussed.

## 1 Introduction

Renewability and sustainability of carbon feedstock are one of the main aims and concerns in metabolic engineering toward producing value-added chemicals. Given the potential to be produced from methane and CO2 (two significant greenhouse gases) (Bertau et al., 2014; Latimer et al., 2018; Marlin et al., 2018), methanol commercially produced from syngas (Clomburg et al., 2017) is appealing as a C- feedstock in terms of environmental carbon sequestration. In addition to the flexible production processes (Schrader et al., 2009), the highly reduced chemical nature of methanol, compared to traditional feedstock like glucose, theoretically allows improved production titers and yields (Whitaker et al., 2015).

**FIGURE 1:**
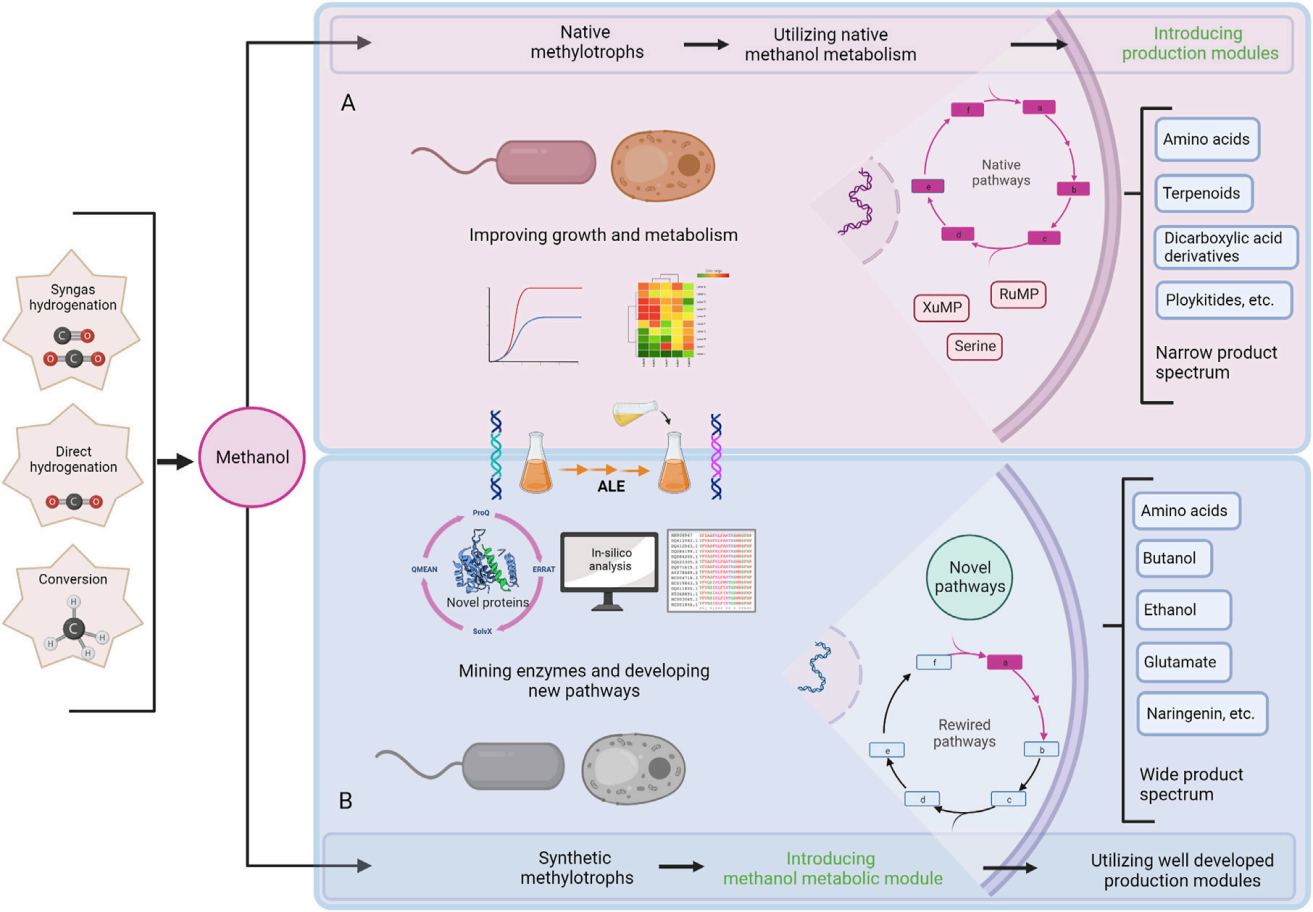
Schematic summary of engineering native and synthetic methylotrophs. **(A)** Engineering scheme of native methylotrophs for chemical production from methanol, **(B)** Engineering scheme of synthetic methylotrophs for chemical production from methanol. ALE; adaptive laboratory evolution.

Methylotrophic bacteria, with the ability to utilize methanol as the sole source of carbon and energy, is one of the potential systems for bioconversion of methanol (Chistoserdova et al., 2009). This group of microbes occurs widely in nature, including both prokaryotes and eukaryotes, and their potential for industrial application was recognized early on. Among the well-studied model methylotrophs, mention might be made of *Methylorubrum extorquens*, *Bacillus methanolicus*, and *Pichia pastoris*, where several chemicals have been targeted for production from methanol. Although various chemicals are produced, inefficiencies in carbon conversion, lack of product diversity, limitations in knowledge of regulatory networks, and updated genetic manipulation tools have been limiting factors for the practical applications of these organisms as an industrial production platform (Zhang et al., 2018). Realizing these shortcomings, efforts have also been made toward synthetic methylotrophy, where well-characterized platform microorganisms with well-studied metabolic networks and advanced engineering tools are targeted for methanol bioconversion (Müller et al., 2015a; Whitaker et al., 2015) (Figure 1). This review will discuss native and synthetic methylotrophs engineered for producing various bulk or value-added chemicals, focusing on engineering tools and techniques utilized therein.

## 2 Methanol metabolism in natural methylotrophs

### 2.1 Oxidation of methanol

Methanol dehydrogenase (Mdh) or alcohol oxidase (Aox) catalyzes the oxidation of methanol to formaldehyde in prokaryotic and eukaryotic methylotrophs (yeast), respectively (Cregg et al., 1989; Sakai and Tani, 1992). Mdh utilizes either pyrroloquinoline quinone (PQQ) in the periplasm or nicotinamide adenine dinucleotide (NAD+) in the cytosol as the terminal electron acceptor for methanol oxidation, depending on whether the organism is gram-negative or thermophilic gram-positive methylotroph, respectively, producing formaldehyde (Anthony, 1982; Krog et al., 2013; Keltjens et al., 2014). Whereas Aox oxidizes methanol in peroxisomes with O2 as an electron acceptor (Pfeifenschneider et al., 2017), generating hydrogen peroxide as a byproduct (Hartner and Glieder, 2006).

### 2.2 Dissimilation of formaldehyde

Due to the highly toxic nature of the metabolic intermediate, formaldehyde (Chen et al., 2016), methylotrophs possess an efficient detoxification mechanism *via* the oxidation of formaldehyde to formate and then CO_2_. Unlike the thiol-dependent formaldehyde dehydrogenases (Flds) of *Escherichia coli* (Gutheil et al., 1992) and *Bacillus subtilis* (Chandrangsu et al., 2014), methylotrophs usually possess tetrahydrofolate (THF)- or tetrahydromethanopterin (H4MPT)-dependent enzymes (Vorholt et al., 1999; Maden, 2000; Yurimoto et al., 2005; Pfeifenschneider et al., 2017) for oxidation of formaldehyde. In methylotrophs, the formaldehyde dissimilation, which is coupled with NADH generation (Chistoserdova, 2011), serves as the primary source of reducing equivalent due to the downregulation of the TCA cycle during growth on methanol as the sole carbon source (Chistoserdova, 2011; Müller et al., 2014; Rußmayer et al., 2015).

### 2.3 Assimilation of formaldehyde

Naturally, methylotrophs follow three main pathways for aerobic formaldehyde assimilation, namely, ribulose monophosphate (RuMP), xylulose monophosphate (XuMP), and serine pathway (Kremp et al., 2018; Tuyishime and Sinumvayo, 2020). In organisms like B. methanolicus and Methylophilus methylotrophus (Schrader et al., 2009), formaldehyde is assimilated *via* the RuMP pathway, where formaldehyde is condensed with ribulose 5-phosphate (Ru5P) by hexulose-6-phosphate synthase (Hps) and then isomerized to form fructose 6-phosphate (F6P) by 6-phospho-3-hexuloisomerase (Phi) (Müller et al., 2015b). In the case of eukaryotic methylotrophs, like P. pastoris, formaldehyde assimilation occurs *via* the XuMP pathway, where a transketolase reaction transfers the glycolaldehyde group of xylulose 5-phosphate (Xu5P) to formaldehyde forming glyceraldehyde 3-phosphate (GAP) and dihydroxyacetone (DHA) (Ro et al., 1997). Both pathways utilize the non-oxidative pentose phosphate pathway (PPP) for anaplerosis, and the assimilation of cellular carbon occurs at the oxidation level of formaldehyde, which is equivalent to that of glucose (Lidstrom, 2006).

In methylotrophs like *M. extorquens*, formaldehyde assimilation occurs *via* the serine pathway (Chistoserdova and Lidstrom, 1994; Ochsner et al., 2015). Unlike the two former pathways mentioned above, here formaldehyde is first converted to formate and then to 5, 10-methylene-tetrahydrofolate (CH2-THF) before incorporating into the pathway using tetrahydrofolate (THF) as the cofactor (Crowther et al., 2008). CH2-THF condenses with glycine (derived from glyoxylate) to form serine; however, (unlike the regeneration of C5-P in RuMP and XuMP *via* PPP pathway) regeneration of glyoxylate occurs through ethylmalonyl-CoA (EMC) pathway circulating the Serine cycle (Erb et al., 2007; Peyraud et al., 2009). While operating the serine cycle, an isotopic labeling study confirmed the incorporation of one CO2, along with the incorporation of each formaldehyde, to form acetyl-CoA (Crowther et al., 2008), pointing toward a dual mode of carbon assimilation.

In the following sections, based on the type of the organism (natural or non-natural methylotrophs) and their route of carbon assimilation, various chemicals, intermediates, and value-added chemicals produced using methanol as a carbon source, will be discussed.

## 3 Targeting natural methylotrophs for chemical production

### 3.1 Utilizing ribulose monophosphate pathway

**FIGURE 2:**
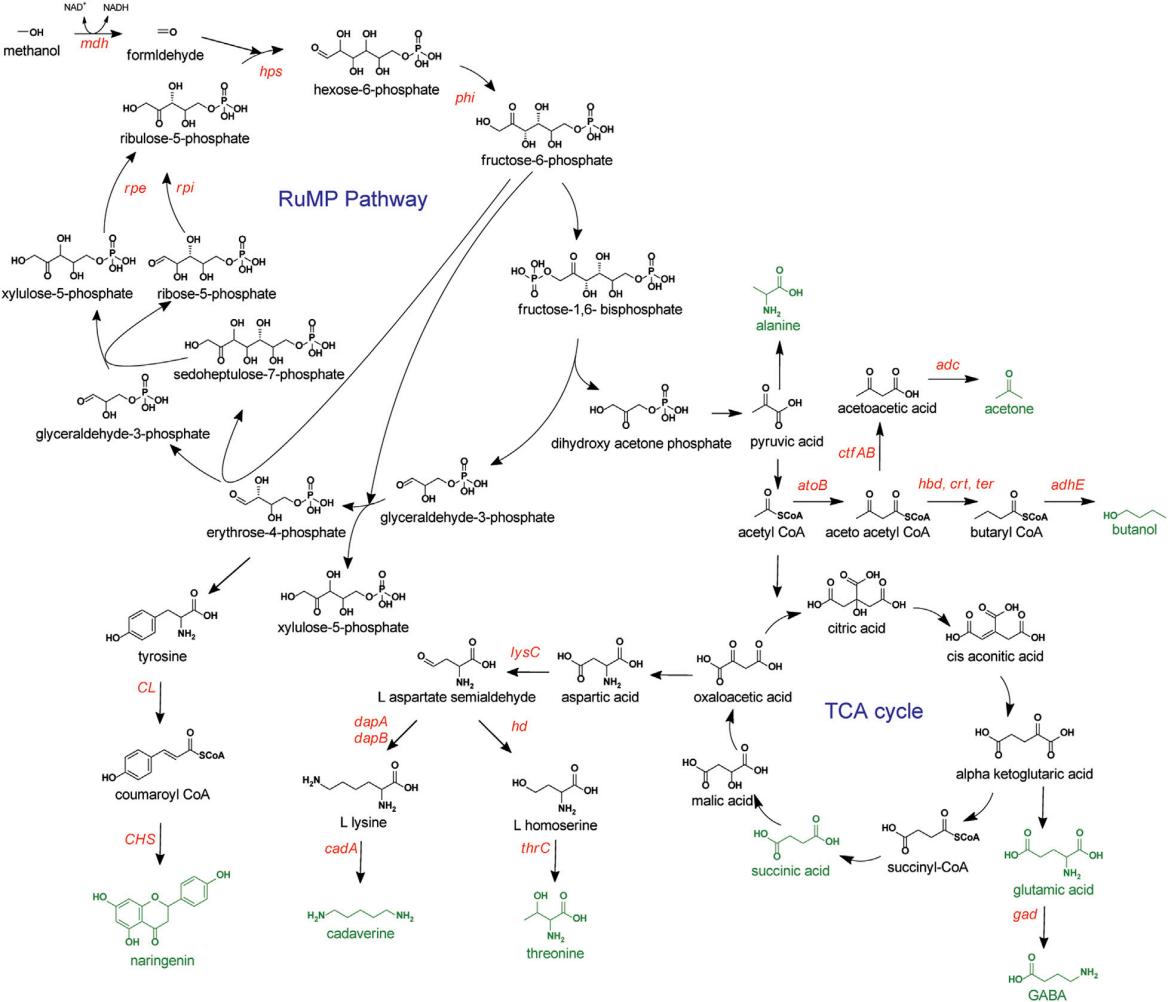
Methanol metabolism *via* RuMP pathway. Compounds targeted for production represented in green. Overexpressed genes or enzymes are presented in red. GABA, gama-aminobutyric acid; Aox, alcohol oxidase; hps, hexulose-6-phosphate synthase; phi, 6-phospho-3-hexuloisomerase; rpe, ribose 5-phosphate epimerase; rpi, ribose 5-phosphate isomerase; CL, 4-coumaroyl CoA ligase; CHS, chalcone synthase; lysC, aspartokinase; dapAB, dihydrodipicolinate synthase and reductase; cadA, L-lysine decarboxylase; hd, homoserine dehydrogenase; thrC, threonine synthase; atoB, acetyl-CoA acetyltransferase; adc, acetoacetate decarboxylase; ctfAB, coenzyme A transferase; hbd, 3-hydroxylbutyryl-CoA dehydrogenase; crt, crotonase; ter, *trans*-2-enoyl-CoA reductase; adhE, aldehyde/alcohol dehydrogenase; gad, glutamate decarboxylase.

The RuMP pathway is considered an efficient route for formaldehyde assimilation in terms of energy consumption and biomass yield since one molecule of ATP and three molecules of NAD(P)H are produced per molecule of pyruvate generation (Chistoserdova et al., 2000). Methylotrophs harboring this pathway have mainly been exploited for the production of amino acids and derivatives (Figure 2). Following are some of the organisms, along with the target chemicals and the strategies.

#### 3.1.1 Production of amino acids and derivatives

##### 3.1.1.1 B. methanolicus

A thermophilic, gram-positive methylotroph, apart from being a model organism for studies of the methylotrophic C-assimilation pathway, has also been widely known for its ability to produce high levels of amino acids. Natural strains of *B. methanolicus* have been reported to produce up to 0.4 g/L L-lysine, 12 g/L L-alanine, 11 g/L L-aspartate, and 59 g/L L-glutamate (Brautaset et al., 2010) from methanol. Mutagenesis and selection studies of the homoserine dehydrogenase gene in these amino acid producing bacteria improved the production of L-lysine and L-glutamate to 65 g/L and 69 g/L, respectively with the supplementation of methionine (Brautaset et al., 2010). The theoretical maximum yield of L-lysine from methanol in *B. methanolicus* has been reported to be similar to that from glucose in industrial amino acid producer *C. glutamicum* (Brautaset et al., 2007). Due to the high production capacity of the above amino acids, *B. methanolicus* was targeted for the production of cadaverine and gamma-aminobutyric acid (GABA), the chemical derivatives of lysine and glutamate, respectively. High cell density fed-batch fermentation using heterologous expression of *E. coli* L-lysine decarboxylase (cadA), which decarboxylates lysine to cadaverine, generated 6.5 g/L of cadaverine (Nærdal et al., 2015). The same group in another study used a two-phase pH shift method to produce 9 g/L GABA by overexpressing glutamate decarboxylase (gad) from *Sulfobacillus thermosulfidooxidans* (Irla et al., 2017).

The plasmid linked methylotrophic nature of these organisms impart high methanol oxidation capacity and tolerance (Brautaset et al., 2004). Together with such high production titers, *B. methanolicus* is a promising candidate as an industrial production platform. Although the thermophilic nature of these organisms may constrain the range of heterologous enzymes or proteins that can be expressed, thermophiles and thermostable enzymes provide unique potential and application in industrial biotechnological processes. Low contamination risk, high reaction rate, and improved substrate solubility are some of the numerous advantages these organisms and enzymes can offer. Methanol fermentation, due to its reduced nature, is high oxygen demanding and generates high heat. The availability of such thermophilic bacteria can help reduce cooling requirements and costs. Additionally, thermostable enzymes like lactate dehydrogenase (Basen et al., 2012), butanol production pathway enzymes (bcs operon) (Bhandiwad et al., 2013), amylases (Lim et al., 2020), etc. that have been expressed for recombinant production of chemicals in thermophiles could be some potential candidates for diversifying the product range in this host.

##### 3.1.1.2 Methylobacillus glycogenes

A gram-negative obligate methylotroph, known for its ability to produce L-glutamic acid, has also been widely studied for amino acid production. In earlier attempts using chemical mutagenesis, different mutant strains producing up to 38.8 g/L L-glutamate, 11 g/L L-threonine, and 3.1 g/L L-lysine were isolated (Motoyama et al., 1993). Furthermore, the high L-lysine production strain was improved by the overexpression of a mutant 4-hydroxy-tetrahydrodipicolinate synthase (dapA) gene partially desensitized to inhibition by L-lysine. The resulting strain was able to produce 8 g/L of lysine from methanol under fermentation conditions (Motoyama et al., 2001). Also, an engineered L-isoleucine auxotroph overexpressing threonine synthase (thrC) increased L-threonine production to 16.3 g/L (Motoyama et al., 1994).

##### 3.1.1.3 M. methylotrophus

Another gram-negative obligate methylotroph *M. methylotrophus* studied for the production of amino acid from methanol. Enzymes involved in the biosynthesis of L-lysine from aspartate, such as aspartokinase (LysC), dihydrodipicolinate synthase (DapA), and dihydrodipicolinate reductase (DapB), were characterized in *M. methylotrophus* and found to be allosterically inhibited by threonine and L-lysine (Gunji et al., 2004). As a follow-up to this finding, LysC, DapA, and DapB from *E. coli*, desensitized to L-lysine feedback inhibition, were overexpressed in an L-lysine analog resistant mutant of *M. methylotrophus*, resulting in a strain that produces 1 g/L L-lysine (Tsujimoto et al., 2006). Furthermore, through overexpression of a mutant L-lysine/L-arginine exporter LysE (insensitive to L-lysine analog) and the mutant DapA (L-lysine insensitive) from *Corynebacterium glutamicum* in wild type *M. methylotrophus*, L-lysine production was improved up to 11.3 g/L (Gunji and Yasueda, 2006). Regarding their successful application, mention might be made of the establishment of *M. methylotrophus* for industrial scale production of single cell proteins (SCP) during the1970s, with production capacity reaching up to 50,000 tons per annum (Windass et al., 1980; Westlake, 1986; Drozd and Linton, 2020). Even though discontinued, owing to the low prices of alternative protein sources, these efforts illustrate their capability as production platforms. These methylotrophic cell factories, in their native or engineered state, exhibit tremendous potential as platform organisms for the industrial production of amino acids and derivatives.

### 3.2 Utilizing serine pathway

**FIGURE 3:**
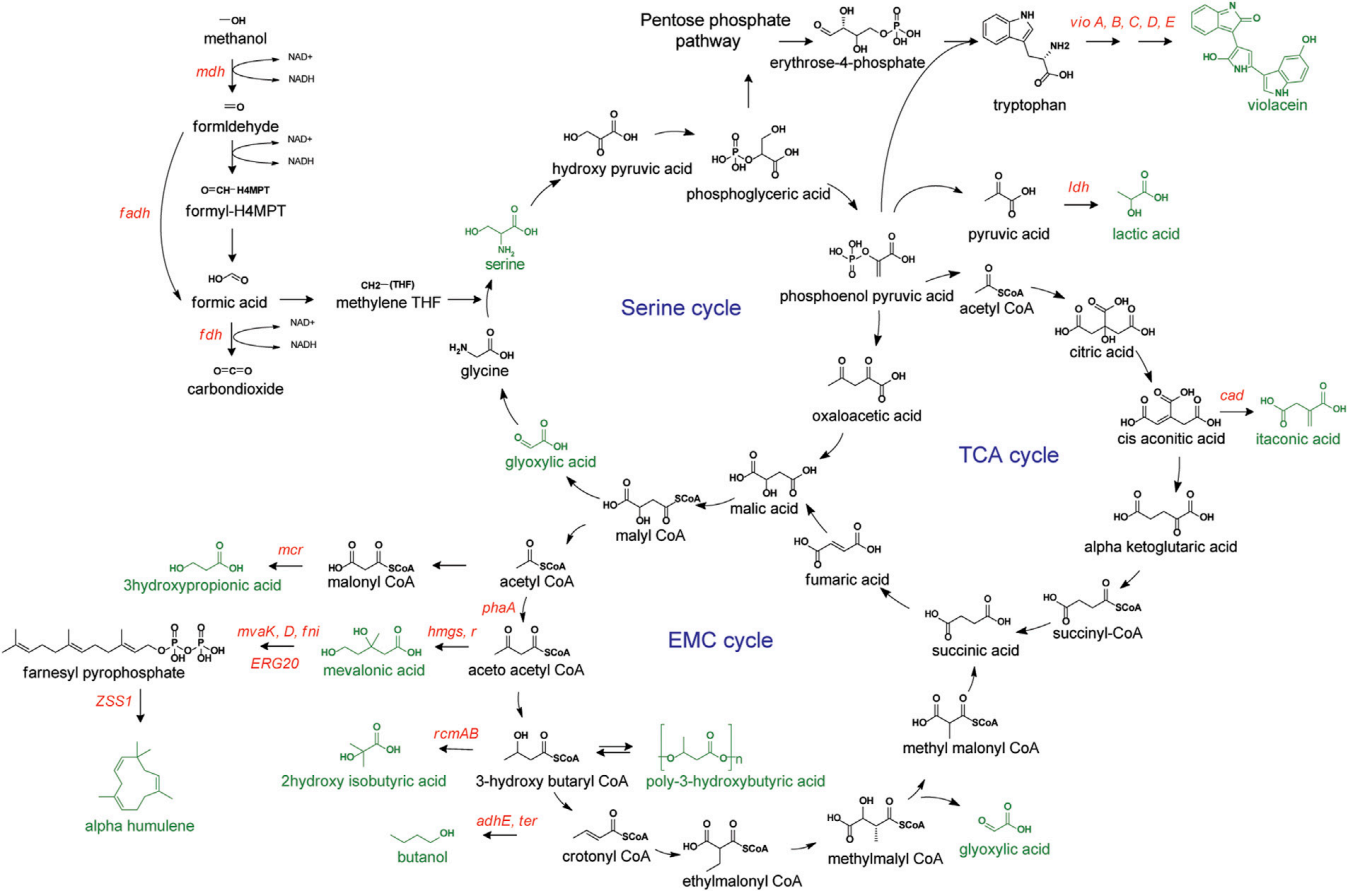
Methanol metabolism *via* Serine pathway. Targeted compounds represented in green. Overexpressed genes or enzymes are presented in red. *mdh*, methanol dehydrogenase; *flds*, formaldehyde dehydrogenase; *fdh*, formate dehydrogenase; *mcr*, malonyl-CoA reductase; *mvaK, D*, mevalonate kinase and decarboxylase; *fni*, isopentenyl-diphosphate delta-isomerase; *ERG20*, farnesyl pyrophosphate synthase; *ZZSl*, α-humulene synthase; *hmgs*, hydroxymethylglutaryl-CoA synthase; *hmgr*, hydroxymethylglutaryl-CoA reductase; *pha*, thiolase; *cad*, *cis*-aconitate decarboxylase; *ldh*, lactate dehydrogenase; *vio A-E*, violacein operon.

Methylotrophs harboring the native Serine pathway operates interlocked alongside the TCA and EMC cycle (Cui et al., 2016). Unlike the RuMP pathway with phosphorylated sugars as intermediates, carboxylic and amino acids are the primary intermediates in the serine cycle, making these organisms suitable hosts for producing chemicals like polyhydroxyalkanoates, and amino and dicarboxylic acids (Figure 3) (Ochsner et al., 2015). *M. extorquens*, one of the most extensively studied model methylotrophs, utilizes serine cycles for formaldehyde assimilation. With the availability of a vast knowledge of physiological, biochemical, and omics information, combined with genome-scale metabolic models, this organism has a promising potential as a methanol-based microbial platform. The following are some chemicals targeted for production from this methylotroph.

#### 3.2.1 Production of amino acids and derivatives

During the methanol assimilation *via* the serine pathway, one glycine molecule reacts with formaldehyde (derived from methanol) to produce L-serine. This very step was utilized for the production of L-serine with glycine as a co-substrate. Using resting cells to prevent glycine toxicity and the freeze-thawing method for facilitating substrate permeability up to 54.5 g/L L-serine was produced (Sirirote et al., 1986). Violacein, a secondary metabolite derived from the shikimate pathway, has also been demonstrated for production from methanol. Overexpression of the violacein biosynthesis pathway genes encoded by the vio operon, consisting of five genes (*vioA, vioB, vioC, vioD*, and *vioE*) caused 11.7 mg/L of violacein production from *M. extorquens* AM1. Subsequently, through directed evolution of the host (for overcoming unidentified bottlenecks) and substrate co-utilization strategy (for increasing supply of reducing power equivalent), a final violacein production titer of 118 mg/L was achieved from methanol and acetate (Quynh Le et al., 2022). Even though the production titer was reportedly lower than that of established *E. coli*, *C. glutamicum*, or *Yarrowia lipolytica* strains (Fang et al., 2015; Sun et al., 2016; Tong et al., 2021), the estimated production cost in terms of substrate and supplement showed substantially lower values with the methylotrophic host.

#### 3.2.2 Production of dicarboxylic acid and derivatives

Methanol dependent growth of *M. extorquens* AM1 is associated with a significant metabolic flux passing through the serine and EMC cycle, generating a steady supply of acetyl CoA. With this acetyl CoA as a precursor, 3-hydroxy propionic acid (3HP) production was attempted. 3HP, a precursor for acrylic acid, and 1,3-propanediol, which could be polymerized to form biodegradable polymers, was targeted for production in *M. extorquens* AM1 through the overexpression of malonyl-CoA reductase (*mcr*) gene of *Chloroflexus aurantiacus* DSM 635 encoding a bifunctional enzyme with alcohol dehydrogenase and aldehyde dehydrogenase activities. Using an adaptively evolved strain of *M. extorquens* AM1 for a faster growth rate (Van Dien and Lidstrom, 2002), a 3-HP titer of 69.8 mg/L was obtained (Yang et al., 2017). To further increase the production titer, increasing the enzymatic activity of Mcr (especially the aldehyde dehydrogenase counterpart of the bifunctional enzyme) along with a sufficient supply of the malonyl-CoA precursor (namely acetyl-CoA) was suggested (Liu et al., 2016). Accordingly, a synergistic methanol assimilation pathway designed by incorporating the RuMP cycle increased the acetyl CoA supply. Expressing *B. methanolicus* derived *hps* and *phi* genes for completing the RuMP cycle and *pfk* for flux distribution from F6P, the engineered strain showed an increased cell growth and methanol consumption rate. Using this newly engineered chassis of *M. extorquens* AM1, 3-HP production was increased up to 0.857 g/L in the fed-batch bioreactor condition (Yuan et al., 2021).

2-Hydroxyisobutyric acid (2-HIBA), a branched C4-hydroxycarboxylic acid, is a precursor of methyl methacrylate, the key intermediate for poly-methyl methacrylate, also known as an alternative glass. An EMC pathway intermediate, 3-hydroxybutyryl-CoA, was employed for the production of 2-HIBA. The coenzyme B12-dependent (R)-3-hydroxybutyryl-CoA mutases (RcmAB) of *Bacillus massiliosenegalensis* JC6 that catalyze the interconversion of (R)-3-hydroxybutyryl-CoA to (R)-2-hydroxyisobutyryl-CoA were overexpressed in *M. extorquens* AM1. By utilizing the poly-3-hydroxybutyrate (PHB) overflow metabolism, the engineered strain was able to produce 2-HIBA to about 2.1 g/L along with PHB as a byproduct, with a maximal combined yield (2-HIBA and PHB) of 0.11 g/g methanol (Rohde et al., 2017).

Although it was a low titer, itaconic acid production has also been demonstrated in *M. extorquens* AM1. Cis-aconitic acid an intermediate of the TCA cycle was decarboxylated with the overexpression of cis-aconitate decarboxylase (Cad) of (*Aspergillus terreus*) resulting in the production of 31.6 mg/L itaconic acid from methanol (Lim et al., 2019). Strengthening the TCA cycle along with its upstream supply chain was suggested as a potential approach for further increasing the production titer.

#### 3.2.3 Production of mevalonate and terpenoids

Mevalonate production was demonstrated in *M. extorquens* AM1 harboring an artificial operon that consisted of the HMG-CoA synthase (HmgS) gene from *Blattella germanica*, the HMG-CoA reductase (HmgR) gene from *Trypanosoma cruzi*, and the thiolase (PhaA) gene from *Ralstonia eutropha*. This engineered strain produced up to 2.2 g L−1 mevalonate with a yield and productivity of 28.4 mg/g and 7.16 mg/L/h under fed-batch fermentation conditions (Zhu et al., 2016). With further media optimization, the concentration, yield, and productivity of mevalonate were increased to 2.59 g/L, 48.90 mg/g, and 12.33 mg/L/h, respectively (Cui et al., 2017). Additionally, with the improvement of acetyl-CoA flux (7%) by using a biosensor-assisted transcriptional regulator (QscR) for increasing the NADPH generation and overexpression of *fumC* (encoding fumarase C), the highest yield of mevalonate (2.67 g/L) in engineered *M. extorquens* AM1 has been reported (Liang et al., 2017). Mevalonate being a precursor of isoprenoid compounds, these strains offers a wide expansion of product spectrum from methanol. Various bulk and value added isoprenoid production strategies established in primary industrial microbes (Navale et al., 2021) could be easily adapted in this host. Relatively, the sesquiterpenoid α-humulene has been successfully produced in *M. extoquens* AM1 by heterologous expression of α-humulene synthase (ZSS1) from *Zingiber zerumbet* and farnesyl pyrophosphate (FPP) synthase from *Saccharomyces cerevisiae* along with isoprenoid precursor supply from mevalonate pathway of *Myxococcus xanthus* (hmgs, hmgr, mvaK1, mvaK2, mvaD, and fni). By using the *crtN* (diapophytoene dehydrogenase) knock-out pigment-less strain of *M. extorquens* AM1 (Van Dien et al., 2003), a final product concentration of up to 1.65 g/L was obtained in two-phase fed-batch cultivation with dodecane as an extraction solvent for avoiding intracellular product accumulation (Sonntag et al., 2015). This production titer of α-humulene is already in competitive range with their production from *E. coli* (Harada et al., 2009). Taking together, all the different chemicals and production stratigies presented above *M. extorquens* AM1provides a reliable platform for the rational engineering of methylotrophic bacteria for industrial application.

### 3.3 Utilizing xylulose monophosphate pathway

The XuMP pathway, natively found in methylotrophic yeast, shares many similarities with the RuMP pathway as far as the metabolic intermediates are concerned. Organisms harboring this pathway might be potentially more efficient in methanol metabolism since the methanol oxidative enzymes are compartmentalized in the peroxisome (confining the toxic intermediates). Like the RuMP pathway, this pathway has also been exploited to produce various chemicals. Model methylotrophic yeast *P. pastoris* is well known for producing recombinant proteins. Apart from being an FDA-approved GRAS (generally recognized as safe) host (Vogl et al., 2013), *P. pastoris* is an attractive platform strain for metabolic engineering because of its high protein secretion efficiency, reduced protein glycosylation, and high cell density culturability (Schwarzhans et al., 2017; Yang and Zhang, 2018). This organism assimilating formaldehyde through the XuMP pathway has been tested to produce various value-added and intermediate compounds.

#### 3.3.1 Production of organic acids

D-Lactic acid, a monomer of the biodegradable polymer PLA (polylactic acid) with high mechanical properties and hydrolysis resistance (Tsuji, 2005), was targeted for production from methanol in *P. pastoris*. D-Lactate dehydrogenase (D-LDH) from *Leuconostoc mesenteroides* was integrated into the rDNA locus *via* a multicopy integrative plasmid and through post transformational gene amplification strategy, leading to engineered strain successfully producing D-lactate (3.48 g/L) under fermentation condition (Yamada et al., 2019). Because of the widespread application of TCA intermediates like fumaric acid, malic acid, and succinic acid in food, chemical, and pharmaceutical industries, and an aim to replace petroleum-derived commodity chemical maleic anhydride, the reductive tricarboxylic acid (TCA) pathway was overexpressed in *P. pastoris*. With the multicopy integration of the pyruvate carboxylase gene (*pc*) and the cytoplasmic malate dehydrogenase gene (*mdh1*) in the genome of *P. pastoris*, Zhang et al., obtained production titers of 0.76, 42.3, and 9.42 g/L for fumaric acid, malic acid, and succinic acid, respectively (Zhang et al., 2015) from a complex medium with methanol supplement. Recently, malic acid production with methanol as a sole carbon source was also attempted. Through an elaborate process involving: 1) expression of heterologous malic acid producing genes along with transporter, 2) knocking out competing pathway (succinic acid and ethanol production pathway), along with methanol dissimilation pathway, and 3) modifying XuMP cycle for optimum methanol assimilation, an engineered *P. pastoris* strain was designed for malic acid production. The final strain produced 2.79 g/L malic acid (Guo et al., 2021).

#### 3.3.2 Production of terpenoids

*P. pastoris* has been used to produce nootkatone, a highly demanded and prized aromatic sesquiterpenoid naturally found in grapefruit, pummelo, or Nootka cypress trees. Initially, a whole-cell biocatalytic system was created by coexpressing the *Arabidopsis thaliana* cytochrome P450 reductase (CPR) and the *Hyoscyamus muticus* premnaspirodiene oxygenase (HPO), which hydroxylated extracellularly added (+)-valencene to (+)-nootkatone. Followed by the introduction of valencene synthase from *Callitropsis nootkatensis* for production of (+)-valencene (Beekwilder et al., 2014). This strain, with the additional overexpression of an endogenous *P. pastoris* ADH and *S. cerevisiae*, truncated Hmg1p (tHMG1) successfully produced 208 mg/L (+)-nootkatone from methanol using dodecane bilayer in a fed-batch bioreactor (Wriessnegger et al., 2014).

#### 3.3.3 Production of polyketide

In an attempt to develop a *P. pastoris*-based *in vivo* fungal polyketide production system, 6-methylsalicylic acid (6-MSA) production was demonstrated by expressing *Aspergillus nidulans* phosphopantetheinyl transferase (PPtase) gene *npgA* and *A. terreus* 6-methylsalicylic acid synthase (6-MSAS) gene *atX* driven by alcohol inducible AOX1 promoter. 6-MSAS, the first fungal polyketide synthase (PKS) gene to be cloned (Dimroth et al., 1976), is one of the best-characterized fungal PKSs. In an initial fed-batch culture, high cell density was achieved (using glycerol as feed), followed by the production of 6-MSA through the methanol induction phase. The strain was able to produce 6-MSA up to 2.2 g/L under methanol induction for 20 h until an intracellular toxic level accumulation of 6-MSA. Promoting higher cell density before methanol induction or developing a fermentation and bio-separation coupled process for releasing 6-MSA were suggested as practical solutions to further increase production levels (Gao et al., 2013).

Anti-hypercholesterolemia pharmaceutical lovastatin (a potent inhibitor of HMG-CoA reductase) and its precursor, monacolin J, have also been produced from *P. pastoris*. Biosynthetic pathway genes for these chemicals from *A. terreus* were strategically assembled to follow a pathway splitting and co-culture strategy. Avoiding the accumulation of pathway intermediates and relieving metabolic stress due to overexpression of complex pathways, a yield of 593.9 mg/L monacolin J and 250.8 mg/L lovastatin were achieved from methanol (Liu et al., 2018a). The lower level of lovastatin was presumably due to the pathway distribution at monacolin J and its partial permeability to the cellular membrane. The lovastatin production was further improved up to 419 mg/L after overexpression of TapA protein, a putative lovastatin efflux pump from the native lovastatin-producing strain *A. terreus* (Liu et al., 2018b).

#### 3.3.4 Production of toxin

Margatoxin (MgTx) is a toxin naturally found in scorpion venom. Due to its high affinity for blocking voltage-gated potassium (Kv) channels, this toxin has been widely used for studying the physiological function of Kv ion channels in various cell types, including immune cells. MgTx maintain the biologically active tertiary structure with three disulfide bonds (Johnson et al., 1994). The chemical synthesis of peptide toxins and production from recombinant *E. coli* require oxidative refolding for proper disulfide bond formation, resulting in low yield (Turkov et al., 1997; Jensen et al., 2009). With the capability to generate high disulfide-rich peptides, the *P. pastoris* expression system was able to produce 83 ± 3 mg/L of fully functional recombinant margatoxin (rMgTx) (Naseem et al., 2021).

Overall, *P. pastoris* represents a promising microbial cell factory for methanol-based bio manufacturing. Compared to the methylotrophic bacteria, the eukaryotic nature of this organism enables higher tolerance to methanol and extreme environmental conditions (pH or temperature), in addition to the possession of protein assembly, folding and post-translational modification systems.

### 3.4 Utilizing other natural pathways

In addition to the common C1 assimilation pathways mentioned above, CO2 assimilation pathways like the reductive acetyl-CoA pathway, Calvin-Benson-Bassham (CBB) cycle, the reductive tricarboxylic acid cycle, the dicarboxylate–4-hydroxybutyrate cycle and reductive glycine pathway (rGLyP) also exist in natural organisms. Among these pathways, the reductive acetyl-CoA pathway is considered the most energetically efficient (Fast et al., 2015) and has been widely explored. The pathway comprises two linear branches for CO and CO2 assimilation: 1) methyl branch, where CO2 is reduced to formate and transferred to tetrahydrofolate (THF), generating formyl-THF. The formyl-THF is further reduced to methyl-THF, which finally transfers the methyl group to acetyl-CoA synthase *via* the corrinoid iron-sulfur protein (CoFeSP). 2) carbonyl branch, where CO or reduced CO2 is directly added to methyl-CoFeSP generated from the methyl-branch producing acetyl CoA. A diverse group of strictly anaerobic facultative autotrophic bacteria knowns as the acetogens utilizes this pathway for carbon assimilation (Ragsdale and Pierce, 2008). Some of these bacteria naturally produce 2, 3-butanediol, acetate, butyrate, ethanol, and butanol (Marcellin et al., 2016). Accordingly, the bio production of chemicals from these organisms using gaseous feedstock (H2/CO2 and CO) has been explored with academic and industrial interest (Liew et al., 2016), and acetone, isopropanol (Berzin et al., 2012; Liew et al., 2022), acetate, ethanol (Berzin et al., 2013), and butyrate (Huang et al., 2019) have been successfully produced from C1 gases. Besides C1 gases, acetogens can metabolize methanol and formate as alternative carbon or energy sources (Drake et al., 1997). In addition to the advantages of transportability, storage, and solubility, methanol and formate offer higher energetic efficiency than the C1 gases (Cotton et al., 2020). Consequently, acetogens cultivated on methanol or formate as the sole carbon source show higher growth rates and production yields in comparison to gaseous substrates, like the production of 12 mM acetate and 3.7 mM butyrate from 20 mM methanol in wild-type *Eubacterium limosum* (Litty and Müller, 2021) and acetate production from formate in *Acetobacterium woodii* (Moon et al., 2021). Introducing the recombinant pathways, butanol and acetone production has also been demonstrated in *E. limosum* with a production of 0.6 mM butanol and 1.6 mM acetone from 100 mM methanol (Flaiz et al., 2021). Considering their flexibility of carbon substrate and conversion efficiency, this group of organisms can very well serve as a platform for chemical production. Nonetheless, their strict anaerobic nature could provide constraints while considering the product spectrum. Due to the focus of this review on methanolic carbon-derived chemical synthesis, the less explored C1 fixing pathways concerning this topic have not been further discussed.

## 4 Engineering pathways for methanol metabolism

Synthetic methylotrophy, the engineering of traditional platform organisms for utilization of C1 compounds by equipping them with methanol-metabolizing modules and pathways, has gained significant interest in recent years (Bennett et al., 2018a). The efforts to engineer methanol utilization in *E. coli*, *C. glutamicum* and *S. cerevisiae* have been reported (Müller et al., 2015a; Witthoff et al., 2015; Dai et al., 2017; Chen and Lan, 2020; Keller et al., 2022). This approach can offer excellent opportunities to utilize the vast synthetic biology tools and pathways already in place in platform organisms and expand the product spectrum of the C1 substrate.

### 4.1 Oxidation of methanol

Engineering the first step in methylotrophy, the oxidation of methanol to formaldehyde, has been the most challenging due to the low catalytic efficiency of MDHs in heterologous systems. Out of the three types of MDHs reported to date, the NAD + - dependent MDH is considered the most suitable candidate as far as synthetic methylotrophy is concerned (Zhang et al., 2017). Unlike the PQQ and O2-dependent MDHs, NAD + -dependent MDHs rely on the ubiquitous cofactor in most platform organisms and are functional under aerobic and anaerobic conditions (Velterop et al., 1995). Several NAD + -dependent MDHs derived from *B. methanolicus* and *B. stearothermophilus* had been tested for methanol oxidation in *E. coli* (Krog et al., 2013; Müller et al., 2015a), with the highest catalytic activity reported from *B. stearothermophilus* (17mU/mg) (Whitaker et al., 2017). In the case of synthetic eukaryotic methylotrophy, FAD-containing AOD from *P. pastoris* has been expressed in *S. cerevisiae* (Dai et al., 2017).

### 4.2 Dissimilation of formaldehyde

Due to its severe toxicity, formaldehyde dissimilation activity is found in most species. Likewise, overexpression of formaldehyde dehydrogenase increased the formaldehyde tolerance of *E. coli* and *S. cerevisiae* (Grey et al., 1996; Kümmerle et al., 1996). Even though proven to increase tolerance, excessive dissimilation can negatively impact carbon yield *via* carbon loss in the form of CO_2_. Therefore, fine-tuning the flux balance between formaldehyde generation and its utilization (assimilation/dissimilation) is a necessity (Chen and Lan, 2020). Dynamic regulation strategies with formaldehyde inducible promoters have demonstrated improved methanol utilization in *E. coli* (Rohlhill et al., 2020). Spatial engineering of separating formaldehyde assimilation and dissimilation modules (naturally occurring in *P. pastoris*) is a plausible strategy for eukaryotic hosts (Rußmayer et al., 2015).

### 4.3 Assimilation of formaldehyde

The most common pathway for engineering methylotrophy in *E. coli* and *C. glutamicum* has been the RuMP pathway; due to the intermediates similarity it shares with the non-oxidative pentose phosphate pathway (PPP). Theoretically, only two enzymes are required to assimilate formaldehyde, i.e., 3-hexulose-6-phosphate synthase (Hps) and 6-phospho-3-hexuloisomerase (Phi). Increasing regeneration of formaldehyde acceptor, Ru5P, by the heterologous introduction of non-oxidative PPP enzymes and deletion of phosphoglucose isomerase (*pgi*) significantly increased methanolic carbon incorporation (Bennett et al., 2018a). Similarly, the intracellular concentrations of Ru5P, F6P, and S7P were significantly enhanced by blocking the lower glycolysis pathway after G3P and over expressing the native bifunctional Fbp/Sbp of *E. coli*, leading to higher formaldehyde assimilation (Woolston et al., 2018a).

Even though considered challenging due to its complexity, introducing serine pathways has also been attempted in *E. coli* for formaldehyde assimilation. The presence of a complete glyoxylate pathway in these organisms could act in favor of circumventing the need for the EMC pathway for glyoxylate regeneration (Yu and Liao, 2018). XuMP pathway introduction in *S. cerevisiae* has been reported for the eukaryotic system.

## 5 Targeting synthetic/non-natural methylotrophs for chemical production

### 5.1 Utilizing synthetic pathways

#### 5.1.1 Production from *E. coli*

Growth or cultivation of *E. coli* utilizing methanol as the sole carbon source has been of limited success; hence, most studies until now rely on a co-substrate feeding strategy (methanol along with a general substrate) for cell growth and biochemical production.

A formaldehyde dehydrogenase (*frmA*) knock-out strain of *E. coli* was developed for the efficient uptake of formaldehyde, overexpressing Mdh of *B. stearothermophilus* and RuMP pathway enzymes Hps and Phi from *B. methanolicus*. The *E. coli* strain successfully produced the valuable compound naringenin using methanol and yeast extract. The introduction of 4-coumaroyl CoA ligase of *A. thaliana* and chalcone synthase of *Petunia hybrida* brought out 3.5 mg/L of naringenin production, where 18% of its carbon was derived from methanol (Whitaker et al., 2017). This recombinant strain was further engineered for enhanced methanol assimilation by incorporating five non-oxidative PPP genes from *B. methanolicus* into the chromosome and deletion of pgi, rerouting the glycolytic carbon flow towards Ru5P through the oxidative PPP. Following the introduction of the 1-butanol production pathway (Kataoka et al., 2015) and the acetone production pathway (Mermelstein et al., 1993) into the resulting *E. coli* strain, successful production of acetone and 1-butanol up to 2.6 g/L and 1.8 g/L, respectively, using methanol and glucose as co-substrates was achieved (Bennett et al., 2018b). However, methanolic carbon incorporation was reported to be very low, about 3.6% in acetone and 0.7% in butanol. The improvement in product titer and the amount of methanol consumption were not well correlated, and metabolite levels, flux profiles, and/or other unknown cellular/metabolic responses were hypothesized for the observed effect. To further increase the methanol co-assimilation, a synthetic methanol auxotrophic strain of *E. coli* was created by blocking essential genes of the PPP, resulting in the co-assimilation of methanol and xylose. The methanol auxotrophic strain was then engineered for the production of ethanol and 1-butanol. An effective increase in methanol utilization was observed with a production titer of 4.6 g/L ethanol and 2.0 g/L 1-butanol with methanolic carbon incorporation of 43% and 71%, respectively (Chen et al., 2018).

Amino acid production is usually associated with high NADPH-demanding synthases (Bommareddy et al., 2014). Incorporating a methanol assimilation module could provide an extra supply of reducing equivalent NADPH. Correspondingly, methylotrophic *E. coli* engineered for lysine production was able to improve production twofold with heterologous expression of NADH kinase from *S. cerevisiae* (Wang et al., 2019a). With NADH identified as a potent kinetic inhibitor of Mdh (Woolston et al., 2018a), this particular step further drives the methanol metabolism in this strain.

#### 5.1.2 Production from *C. glutamicum*

*C. glutamicum*, a well-studied and preferred industrial workhorse, has also been targeted for methanol metabolism. By expressing Mdh from *B. methanolicus*, along with Hps and Phi, in a strain devoid of endogenous aldehyde dehydrogenase genes *ald* and *fadH*, a methylotrophic *C. glutamicum* was engineered, and cadaverine production was attempted. Using 13C methanol and either glucose or ribose as a co-substrate, 1.5 g/L of cadaverine with 5%–15% labeled carbon was produced from this strain (Leßmeier et al., 2015). In another study, a methanol-dependent *C. glutamicum* strain created *via* ribose 5-phosphate epimerase (*rpe*) knock-out, equipped with Mdh from *B. methanolicus*, and Hps and Phi from *B. subtilis*, has been tested for cadaverine production. Combining the methanol-dependent complementation of this metabolic cutoff strain with ALE (Adaptive Laboratory Evolution) for increasing methanol dependency, the final strain produced 163 mg/L cadaverine with 43% labeled carbon using 13C-methanol and ribose as carbon substrates (Hennig et al., 2020). Another methanol-dependent *C. glutamicum*, a ribose 5-phosphate isomerase (*rpi*) deficient mutant, with Mdh of *B. stearothermophilus* and RuMP enzymes (Hps and Phi) of *B. methanolicus* has been engineered for production of glutamate. In a minimal medium supplemented with methanol and xylose (4 g/L each), the strain produced 90 mg/ml glutamate (Tuyishime et al., 2018). Further increasing the methanol tolerance with ALE the production titer was improved up to 230 mg/L (Wang et al., 2020). As a native glutamate producer, these low production titers indicate poor methanol assimilation and metabolism; developing strategies for enhancing methanol tolerance and incorporation are the current challenges for this host.

#### 5.1.3 Production from *S. cerevisiae*

In addition to the native methylotrophic yeast *P. pastoris* discussed above, attempts have been made to introduce methylotrophy in *S. cerevisiae*. Compared to the prokaryotic bacterial platforms, *S. cerevisiae* possesses great potential as a host for introducing methylotrophy due to its high methanol tolerance (Yasokawa et al., 2010). By integrating the methanol oxidation pathway of *P. pastoris* into the chromosome, a recombinant *S. cerevisiae* strain capable of growth on methanol as a sole carbon source was developed. The recombinant strain consumed up to 1 g/L methanol, producing 0.26 g/L pyruvate (Dai et al., 2017). Additionally, the native capacity of methanol utilization has also been realized in *S. cerevisiae* (Espinosa et al., 2020). For further advancement, it may be worthwhile to investigate how this capability works in conjunction with the optimization of the expression strength and balance of heterologous methylotrophic pathway enzymes.

### 5.2 Utilizing novel synthetic pathways

#### 5.2.1 Modified serine pathway

**FIGURE 4:**
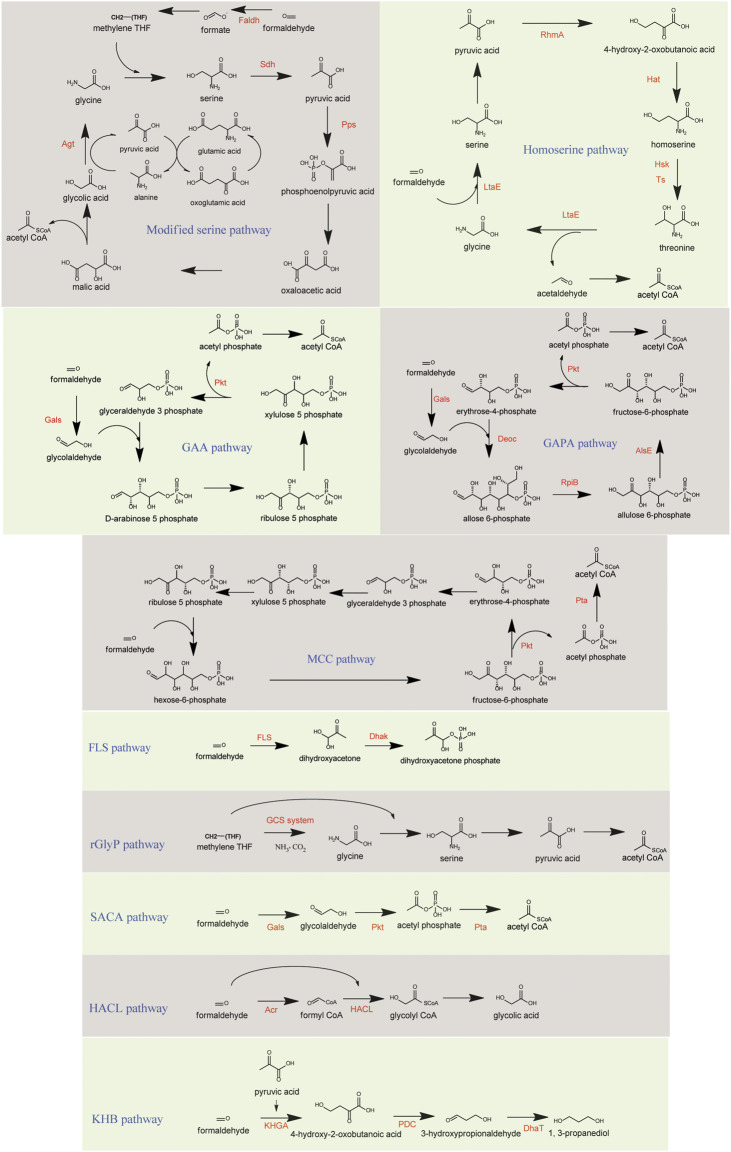
Methanolic carbon assimilation in synthetic pathways. Key enzymes are presented in red. GAA, glycoaldehyde assimilation pathway; GAPA, glycolaldehyde-allose 6-phosphate pathway; MCC, methanol condensation cycle; FLS, formolase pathway; rGlyP, reductive glycine pathway; SACA, synthetic acetyl-CoA pathway; HACL, 2-Hydroxyacyl-CoA lyase pathway; KHB, ketohydroxy butyrate pathway. Faldh, formaldehyde dehydrogenase; Sdh, serine dehydratase; Pps, phosphoenolpyruvate synthase; Agt, alanine-glyoxylate transaminase; LtaE, threonine aldolase; RhmA, 2-keto-3-deoxy-L-rhamnonate aldolase; Hat, HOB aminotransferase; Hsk, homoserine kinase; Ts, threonine synthase; Gals, benzoylformate decarboxylase; Pkt, phosphoketolase; DeoC, 2-deoxy-D-ribose-5-phosphate aldolase; RpiB, ribose-5-phosphate isomerase B; AlsE, D-allulose-6-phosphate 3-epimerase; Pta, phosphate acetyltransferase; FLS, formolase; Dhak, dihydroxyacetone kinase; GCS system, Glycine cleavage system; Acr, acetyl CoA reductase; HACL, 2-hydroxyacyl-CoA-lyase; KHGA, 2-keto-4-hydroxyglutarate aldolase; PDC, pyruvate decarboxylase; DhaT, 1,3-propanediol oxidoreductase.

In this pathway, the oxidation of formaldehyde to formate is achieved in a single step catalyzed by heterologous Faldh from *P. putida*, simplifying the four-step process in the native serine cycle. Additionally, the hydroxypyruvate reductase (Hpr) route was avoided, where native Hpr exhibit higher catalytic activity for the irreversible conversion of intermediate glyoxylate to glycolate rather than converting hydroxypyruvate to glycerate. Instead of employing serine as an amino group donor, glyoxylate is transaminated with alanine to generate glycine in a reaction catalyzed by *S. cerevisiae* alanine-glyoxylate transaminase (Agt). Glycine accepts MTHF to generate serine which is finally deaminated to pyruvate through serine dehydratase (Sdh) from *Cupriavidus necator*. PEP is regenerated by the action of endogenous phosphoenolpyruvate synthetase (Pps), thus avoiding the formation of the intermediate hydroxypyruvate (Figure 4). *E. coli* equipped with this pathway reported to produce 2.2 g/L acetate and 1.6 g/L ethanol with methanol and xylose as co-substrates. 13C-labeling results showed methanol assimilation into acetate and ethanol at 38% and 25%, respectively (Yu and Liao, 2018).

#### 5.2.2 Homoserine pathway

Another derivative of the serine cycle is designed to avoid the competition of pathway flux with the central metabolism, increasing thermodynamic favorability and avoiding energy-expensive carboxylation. In this cycle, the promiscuous activity of *E. coli* threonine aldolase (LtaE) catalyzes the direct condensation of glycine with formaldehyde to generate serine. Serine is directly converted to pyruvate, bypassing formaldehyde condensation to the CH2-THF route of the serine cycle, which follows the condensation of pyruvate with formaldehyde generating 4-hydroxy-2-oxobutanoate (HOB) *via* the promiscuous activity of *E. coli* 2-keto-3-deoxy-L-rhamnonate aldolase (RhmA). HOB is subsequently aminated to homoserine by HOB aminotransferase (Hat). Homoserine is later metabolized by homoserine kinase (Hsk) and threonine synthase (Ts) to produce threonine. Finally, the activity of LtaE cleaves threonine producing acetaldehyde and regenerating glycine (Figure 4). Acetaldehyde, thus produced, is further oxidized to acetyl-CoA (He et al., 2020). Using *E. coli* auxotrophic strains, the functionality of this cycle was demonstrated *in vivo* (He et al., 2020).

#### 5.2.3 Methanol condensation cycle

Methanol condensation cycle (MCC) is a RuMP pathway derived carbon conserved and ATP-independent synthetic biocatalytic pathway that uses enzymatic reactions of the non-oxidative glycolysis (NOG) to convert formaldehyde to acetyl-CoA and water (Bogorad et al., 2013; Bogorad et al., 2014). Similar to the RuMP cycle, formaldehyde is combined with Ru5P to form Hu6P, which is further isomerized to F6P. Unlike the RuMP cycle, where F6P is phosphorylated to FBP and later cleaved to GAP and DHAP, in the MCC, the phosphoketolase (Pkt) cleaves F6P to acetyl phosphate and erythrose 4-phosphate. The acetyl phosphate can be readily converted to acetyl-CoA by a phosphate acetyltransferase (Pta), and erythrose 4-phosphate is used to regenerate Ru5P (Figure 4). This pathway can increase the carbon yield by avoiding the decarboxylation of pyruvate for acetyl-CoA formation accompanying CO_2_ release (Bogorad et al., 2014). Using this pathway, 610 mg L−1 ethanol and 170 mg L−1 butanol production were demonstrated *in vitro*.

#### 5.2.4 Glycolaldehyde assimilation pathway

Another novel C1 assimilation pathway based on phosphoketolase (Pkt) was developed through the *in silico* combinatorial algorithm called comb–flux balance analysis (Yang et al., 2019). In this multienzyme system, two formaldehyde molecules are first condensed to glycolaldehyde by an engineered benzoylformate decarboxylase (Gals) which behaves like an aldolase. Subsequently, glycoaldehyde condenses with glyceraldehyde-3-phosphate (G3P) to form Ru5P, which is isomerized to Xu5P. Finally, Pkt cleaves Xu5P generating G3P and producing the acetyl-CoA precursor acetylphosphate (Figure 4). *In vitro* demonstration of this pathway showed the production of 462.6 mg/L acetate after pathway optimization (Yang et al., 2019).

#### 5.2.5 Glycolaldehyde-allose 6-phosphate pathway

Similar to the GAA pathway and developed by the same group, the glycolaldehyde-allose 6-phosphate (GAPA) pathway condenses the formaldehyde-derived glycoaldehyde with E4P generating 2R, 3R-stereo allose 6-phosphate (A6P), catalyzed by the novel aldolase DeoC. A6P is then isomerized to d-allulose 6-phosphate (Au6P) by allose 6-phosphate isomerase/ribose 5-phosphate isomerase B (RpiB) and subsequently epimerized to F6P by D-allulose-6-phosphate 3-epimerase (AlsE). Finally, F6P is hydrolyzed by Pkt to produce acetylphosphate regenerating E4P (Figure 4). With this pathway, the *in vitro* carbon yield was further improved (94%) compared to the GAA (88%) (Mao et al., 2021).

#### 5.2.6 Formolase pathway

The formolase pathway is a novel, computationally designed carbon fixation pathway. The *de novo* engineered enzyme formolase (FLS), catalyzing the carboligation of three molecules of formate to a DHA molecule (Siegel et al., 2015), was used to incorporate carbon into the central metabolism (Figure 4). Combining the expression of NAD-dependent Mdh from *B. methanolicus* with formolase and native dihydroxyacetone kinase (Dhak), a linear pathway for methanol assimilation was demonstrated for the first time in *E. coli* (Wang et al., 2017). Despite being unable to grow exclusively on methanol, the strain displayed greater methanolic carbon incorporation in the proteinogenic amino acids compared to the parental strain after adaptive evolution for improved methanol utilization.

#### 5.2.7 Reductive glycine pathway

The Reductive Glycine Pathway (rGlyP) is a synthetic pathway, which theoretically provides the most energy-efficient formate assimilation route. This pathway starts with the ligation of formate with H4F and further reduces to CH2-THF. Then glycine cleavage/synthase system (GCS) reacts CH2-THF with CO2, ammonia, and NADH, generating glycine. Following this, another CH2-THF condenses with glycine to form serine. The pathway starting from formate up to serine is fully reversible, and the direction of metabolic flux is determined by the concentration of substrates and products (Bar-Even et al., 2013). This pathway was successfully established in *E. coli*, creating strains able to grow on formate as the sole carbon source (Yishai et al., 2017; Kim et al., 2019). Additionally, with the introduction of Mdh from *Pseudomonas putida*, a short-term ALE evolved strain capable of utilizing methanol *via* this pathway has been reported (Kim et al., 2020). The engineered strain grew on methanol and CO2 with a doubling time of 54 ± 5.5 h and a biomass yield of 4.2 ± 0.17 gDCW/mole methanol. rGlyP has also been functionally demonstrated in *S. cerevisiae* (Gonzalez de la Cruz et al., 2019).

#### 5.2.8 Synthetic acetyl-CoA pathway

The synthetic acetyl-CoA (SACA) pathway is a linear C1 assimilation pathway where two formaldehyde molecules are condensed to glycolaldehyde catalyzed by a glycolaldehyde synthase (Gals). Subsequently, an Actinobacteria-derived Pkt with acetylphosphate synthase activity converts the glycolaldehyde to acetylphosphate. Finally, acetyl-CoA is generated *via* the action of Pta (Figure 4). Although theoretically one of the thermodynamically favorable pathways, the enzymes involved (Gals and Pkt) displayed very low substrate affinities *in vivo*. The *in vitro* demonstration of this pathway by 13C-labeled metabolites achieved a carbon yield of ∼50%; however, when introduced to *E. coli*, only 17% average carbon labeling was detected in PEP (Lu et al., 2019).

#### 5.2.9 2-Hydroxyacyl-CoA lyase pathway

The 2-Hydroxyacyl-CoA lyase (HACL) pathway is another C1 assimilation pathway based on the activity of 2-hydroxyacyl-CoA-lyase (HACL). This enzyme can act reversibly and catalyze the ligation of carbonyl-containing molecules with formyl-CoA to produce C1-elongated 2-hydroxyacyl-CoAs (Cotton et al., 2020). In this pathway, formaldehyde is converted to formyl-CoA *via* an acyl-CoA reductase, and the HACL catalyzes the ligation of formaldehyde with formyl-CoA to generate glycolyl-CoA, which can be converted to glycolate (Figure 4). Engineered *E. coli* whole-cell biocatalyst ultimately produced 1.2 g/L of glycolate in 24h from formaldehyde (Chou et al., 2019).

#### 5.2.10 Ketohydroxybutyrate pathway

Ketohydroxybutyrate (KHB) pathway is a pyruvate-based formaldehyde assimilatory pathway utilizing the aldol condensation activity of the natural enzyme 2-keto-4-hydroxyglutarate (KHG) aldolase. In this pathway, formaldehyde condenses with pyruvate to form HOB, which can be further decarboxylated *via* alpha-keto acid decarboxylase to 3-hydroxypropionaldehyde (3-HPA) and finally reduced to a value-added product 1, 3-propanediol (1, 3-PDO) using the enzyme 1, 3-PDO oxidoreductase. Engineered *E. coli* expressing this pathway successfully demonstrated the production of 32.7 mg/L 1, 3-propanediol from methanol with glucose supplement (Wang et al., 2019b).

All the aforementioned novel pathways have been designed to make effective use of endogenous cellular resources during methanol metabolism. Although functionally illustrated as proof of concept *in vitro* and/or *in vivo* for the assimilation of C1 substrates, these novel engineered pathways are still far from optimum. The difficulties of increasing enzyme efficiencies, efficient metabolic rerouting, and regulation of host metabolism are still hurdles to overcome for transforming synthetic methylotrophs into a methylotrophic platform organism.

## 6 Conclusion and future prospects

The urgency to reduce carbon emissions was reaffirmed at the COP26 summit. Using C1 compounds as a feedstock for the bio-production of commodity chemicals could offer a sustainable solution. Methanol, being a renewable and scalable C1 feedstock, has attracted considerable attention. Consequently, developing methylotrophic microbial platforms could potentially offer a sustainable bio-economy. As a result, research on natural and synthetic methylotrophy has garnered increasing interest from the scientific community. Both approaches to methylotrophy have had significant improvements in a short period, even though not yet up to the potential for a viable industrial-scale application. Native methylotrophs, although with the innate methanol utilization capability, could not be utilized to their full capacity as a platform organism due to insufficient knowledge of the intricate regulatory networks and mechanisms governing the metabolism, in addition to the lack of well-developed genetic engineering tools. Acknowledging these gaps, mention might be made of some recent advances with the potential implications to bridge them, like the identification of a total of 147 genes indispensable for methylotrophy in *M. extorquens* AM1, along with the essential regulatory role of the phosphoribulokinase gene (Ochsner et al., 2017). Development of biosensor-assisted transcriptional regulators for control metabolic flux re-distribution (Liang et al., 2017) and Cre/loxP systems for heterologous pathway integration into the genome of AM1 (Liang et al., 2018) are some invaluable assets. Inducible/regulated expression of recombinant genes in methylotrophs has been another long-realized necessity. The regulatory elements of *Pseudomonas putida* F1 have been utilized to develop a tightly regulated inducible promoter system in AM1 (Choi et al., 2006). The methylotrophic promoter tool set was further upgraded by the development of a variety of inducible and tunable promoters of a wide expression range (Carrillo et al., 2019; Sathesh-Prabu et al., 2021). Also, utilizing the repABC regions for extrachromosomal DNA maintenance, minichromosomes with stable inheritance compatible with other plasmid systems have been developed for AM1 (Liang et al., 2018). The microbial physiology and plasmid stability of AM1 under high cell density fed batch culture have also been investigated to assess their industrial applicability (Bélanger et al., 2004).

As for the synthetic methylotrophs, even with the vast knowledge and available tools, tolerance and efficient utilization of methanol with sufficient regeneration of pathway intermediates for incorporation of methanol-derived carbon remain a challenge. Techniques like improvement of substrate uptake and reduction of methanol toxicity (Meyer et al., 2018), characterization and evolution of new and novel alcohol dehydrogenase (Wu et al., 2016), surveying pathway bottlenecks through isotopic tracing (Woolston et al., 2018b), and utilizing advanced bio-computational tools for predicting enzyme and pathway kinetics (Woolston et al., 2018b) are some promising approaches adopted to meet these challenges. Among the model organisms studied to endow methylotrophy, *E. coli* garnered the most attention with successful production of succinic acid (Zhang et al., 2018), ethanol, and 1-butanol (Chen et al., 2018) from methanol and other co-substrates. However, for these strains to be of practical relevance, the growth and methanolic carbon incorporation requires significant enhancement. Through rational designing and extensive ALE techniques, *E. coli* with doubling time (8.5 h and maximum OD of 2) and methanol tolerance (up to 1.2 M) comparable to native methylotrophs have been reported (Chen et al., 2020).

Irrespective of their present status, both the methylotrophy approaches follow a parallel track in the pursuit of bioprocess from a renewable alternative carbon feedstock.
